# Helpers' stories of providing support to bereaved individuals following drug-related death: a narrative study

**DOI:** 10.1080/17482631.2026.2650824

**Published:** 2026-03-27

**Authors:** Cecilie Kristiansen, Vibeke Samsonsen, Sari Kaarina Lindeman

**Affiliations:** a Bjørgvin Family Office, Bergen, Norway; b Western Norway University of Applied Sciences, Bergen, Norway

**Keywords:** Focus group, helper, bereaved, drug-related death, service development, recognition

## Abstract

**Purpose:**

Drug-related death is a serious public health issue affecting many bereaved families. Drug-related deaths are defined as fatalities resulting directly from the consumption of narcotics, where the cause is violence, accidents, or other health disorders associated with drug use. Individuals bereaved by drug-related death represent a vulnerable group who may experience grief, shame, stigma, and a lack of support from society.

**Methods:**

This article draws on a focus group study involving 29 helpers, examining their narratives about helpful support for individuals bereaved by drug-related death.

**Results:**

The study highlights four key themes describing helpful support: the accessible helper, the empathetic, acknowledging helper, the caring, present helper, and the self-caring helper.

**Discussion:**

Helpers emphasise the importance of being accessible and responsive, yet they face significant barriers in reaching bereaved individuals, often stemming from stigma and a lack of recognition of their needs. They underscore the value of compassionate engagement and the need for peer support to navigate their roles effectively and prevent burnout. Despite their awareness of these factors, the current support guidelines are not adequately integrated into municipal services, indicating a crucial need for further research and improvements.

## Introduction

Deaths caused by problematic drug use are a global concern (European Monitoring Centre for Drugs and Drug Addiction, 2024), impacting numerous bereaved family members and friends. In Norway, approximately 300 people die annually from drug-related causes (Norwegian Institute of Public Health, 2021). In this article, drug-related deaths are defined as fatalities resulting directly from the consumption of substances classified as narcotics, as well as deaths among individuals who use narcotics where the cause is violence, accidents, infectious diseases, or other health disorders that may be indirectly associated with drug use. The term “bereaved individuals following drug-related death” refers to individuals who have lost someone close—such as a family member, friend, or loved one—due to drug-related death. These individuals often experience severe grief reactions a long time after the death loss and grief caused by the death of someone involved in drug use and struggle with complex relationships, stigmatisation, intense emotions, and social isolation (Kalsås et al., 2023; Kalsås, 2024; Titlestad et al., 2021).

In Norway, guidelines for supporting bereaved individuals are outlined in the Norwegian Directorate of Health's (2017) national recommendations for sudden death. These guidelines state that bereaved individuals should receive adequate emotional support, structured follow-up, and continuity in the helpers they interact with. According to the guidelines, children should be involved in the support process, and bereaved individuals should be informed about typical grief reactions. It is also noted that sudden deaths, such as suicide and overdoses, are often stigmatised and associated with feelings of shame and guilt. However, engaging in conversations with bereaved individuals can help alleviate these negative emotions (Norwegian Directorate of Health, 2017). Nevertheless, as drug-related deaths have not been explicitly addressed in the guidelines, this group of bereaved individuals may have been overlooked in relation to psychosocial follow-up from municipal services (Reime & Dyregrov, 2022).

Globally, there is limited research on the experiences and perspectives of professionals supporting bereaved individuals following drug-related deaths, as highlighted in the systematic review by Reime et al. (2022). This gap underscores the need for further examination of these professionals' viewpoints to enhance education and improve the quality of health and social services, as well as to raise awareness about the needs of those grieving after drug-related fatalities (Løseth and Selseng, 2022; Valentine et al., 2018).

Research on service providers' voices is essential for understanding how their experiences and insights influence the quality and relevance of services, such as helpers providing grief support in both the short and long term within municipalities, and other relevant actors who come into contact with bereaved individuals after drug-related deaths. This is also aligned with the principles of evidence-based practice (Connor et al., 2023), which combines research-based knowledge, practical experience, and user perspectives to inform decision-making processes in service delivery. By being on the front lines, service providers maintain direct contact with bereaved individuals, which equips them with valuable knowledge about their needs and effective support practices.

Overall, there is a clear need for enhanced knowledge regarding the experiences of professional helpers. Insights into the practices, challenges, and opportunities they face can be valuable in supporting vulnerable populations encountering various health and social challenges. Specifically, when it comes to supporting bereaved individuals after drug-related deaths, these insights should be considered within broader contexts, including societal norms, cultural values, and the legal and policy landscapes, to fully address the complexities involved (cf. Løseth and Selseng, 2022).

In this work, we aim to bridge the knowledge gap by exploring professional helpers' stories of supporting bereaved individuals. Specifically, the study seeks to understand what helpers identify and give meaning to as helpful in supporting those bereaved by drug-related deaths. This research focuses on the narratives of professional helpers who routinely support bereaved individuals following such losses.

The following research question guides our exploration:

How do professional helpers describe the key factors in providing helpful support to bereaved individuals?

## Previous research

The life situation of bereaved individuals following drug-related deaths was long an under-researched area (Titlestad, Lindeman, et al., 2021), but in the past five years the field has been given more attention (e.g., Kalsås et al., 2024; Lambert et al., 2022; Meen et al., 2024; O'Callaghan, 2024; Stout & Fleury-Steiner, 2023; Titlestad et al., 2025). The research on bereaved people’s experiences of grief processes after drug-related deaths encounters a myriad of stressors, including complex relationships (Kalsås et al., 2024) and stigmatisation (Dyregrov & Selseng, 2022; Stout & Fleury-Steiner, 2023), complicated emotions such as shame, guilt, and relief (Lambert et al., 2022; Titlestad, Mellingen, et al., 2021), along with social isolation and difficulties communicating about their loss (Kalsås et al., 2023).

Research on the bereaved individuals’ experiences with professional help indicates that those bereaved by drug-related deaths often do not receive adequate support during their grief process (Kalsås et al., 2023; Lambert et al., 2022; O'Callaghan, 2024; Reime et al., 2024). Recent studies highlight that bereaved individuals require support and follow-up after the loss of a loved one, similar to others who have experienced sudden and unexpected loss (Kalsås et al., 2023; Lambert et al., 2022). However, studies suggest these individuals often do not receive necessary support due to barriers such as a lack of availability of services and competencies (e.g., Kalsås et al., 2023; Templeton et al., 2016; Titlestad & Dyregrov, 2022), as well as the lack of recognition of this group of bereaved (Løseth and Selseng, 2022) or the stigma towards the deceased and the bereaved individuals (Dyregrov & Selseng, 2022; Stout & Fleury-Steiner, 2023). In a study by Kalsås et al. (2023), only half of the bereaved children following drug-related deaths received any support, and of those, only half were satisfied with what they received.

Bereaved individuals face a high risk of prolonged grief and trauma responses, which can negatively impact their health, quality of life, and ability to function (Titlestad & Dyregrov, 2022). Many express a strong need to be recognised by the support system as a distinct group (Templeton and Yarwood, 2018), and this also applies to younger individuals (Kalsås et al., 2023). However, with adequate support, they also experience better coping with grief and post-traumatic growth (O’Callaghan et al., 2023; Titlestad et al., 2022). According to evidence-based practices, support should be tailored to the specific needs and preferences of the bereaved (Kalsås et al., 2023). Continuity in help is deemed essential, as bereaved individuals benefit from long-term support from helpers (Titlestad & Dyregrov, 2022). Moreover, helpers should approach the bereaved with appropriate language, kindness, respect, and knowledge regarding traumatic loss (Cartwright, 2019; Walter et al., 2017). As noted by Dyregrov & Dyregrov, (2008) in their book on sudden death bereavement, the quality of support is critical. Support services often lack sufficient resources and expertise to provide adequate support, highlighting the need for improvement through political awareness, education, and training (Kalsås et al., 2023).

Current research from the helpers' perspective remains limited (Reime et al., 2024; Totland et al., 2024; Valentine et al., 2018), but essential insights have emerged from studies like Løseth and Selseng, 2022; 2023) in Norway, which highlight that bereaved individuals following drug-related deaths are often excluded from crisis services and psychosocial follow-up. Moreover, very few municipalities have established routines for applying national guidelines in cases of drug-related death, and many helpers reported that critical information did not reach the appropriate services, preventing follow-up measures (Løseth and Dyregrov, 2023; Løseth, Selseng, et al., 2023; Løseth and Selseng, 2022). Lastly, helpers may require various forms of self-support when working with bereaved individuals following drug-related death to prevent secondary trauma (O'Callaghan & Lambert, 2024; Reime et al., 2024).

## Methodology

This study is part of the END project, a large Norwegian cross-sectional, mixed-methods study (2017–2024), looking at the experiences of individuals bereaved by drug-related deaths, their psychosocial health, their encounters with support services, and how they are addressed by professional services (Western Norway University of Applied Sciences, 2020). The present study employs a qualitative design. Data was collected through semi-structured focus group interviews. Thematic narrative analysis (Riessman, 2008) was used to explore the narratives of 29 professional helpers about providing helpful support to bereaved individuals following drug-related deaths. Due to the scarcity of previous research in this field, the study employs an exploratory design and bases its analysis on empirical data.

### Participants and recruitment

Participants were recruited from six Norwegian municipalities selected based on their high levels of drug-related deaths. These target municipalities, varying in population size and structure, were identified and contacted through the Norwegian Directorate of Health's pilot project addressing high overdose mortality rates. Recruitment was conducted using the snowball sampling method (cf. Biernacki & Waldorf, 1981). We acknowledge that although recruitment was initiated through multiple gatekeepers in each municipality, the use of snowball sampling may still have concentrated participants within overlapping professional networks. This may have limited the diversity of perspectives represented. To mitigate this, we actively sought participants across services and hierarchical levels. Eligible professional helpers were those who interacted with bereaved individuals through municipal health and welfare services or voluntary organisations. To reduce reliance on a single internal network and broaden the pool, recruitment was initiated through multiple entry points in each municipality (local project leads, municipal coordinators, and voluntary organisations), and initial contacts were asked to recommend colleagues from different services and hierarchical levels. We acknowledge that snowball sampling may still have led to some concentration within local professional networks.

Recruitment of professional helpers for the focus group interviews began in spring 2019. Through informed consent, participants were given details about the length of the interviews, the audio recording and transcription process, and the voluntary nature of participation.

The sample consisted of 29 professional helpers, including eight men and 21 women, representing the following professions: social worker, doctor, child welfare officer, learning disability nurse, public health nurse, and psychologist. Participants were drawn from various services, including social services (3), grief therapy (1), general health services (1), local mental health and drug services (8), residential care services (5), low threshold support services for persons with drug use (6), next of kin services (2), services for children, youth, and families (2), and specialised drug addiction and rehabilitation services (1).

### Focus group interviews

A semi-structured, pre-piloted thematic guide was used to conduct the focus group interviews. The guide contained questions regarding the helpers' experience with grief support following drug-related deaths, challenges encountered, examples of best practices, collaboration experiences, and the need for support. Three teams, each consisting of two researchers, were responsible for conducting interviews in the six municipalities. One of these teams was led by the third author. Focus group interviews were held in meeting rooms either at the participants' workplaces or nearby, convenient locations with the necessary facilities. The focus groups each contained two to seven participants, and the interviews lasted between 1.5 and 2.5 hours, depending on the size of the group. The interviews were conducted from September 2019 to January 2020.

### Thematic narrative analysis

Narrative analysis is used to examine social processes and how individuals use stories to construct meaning, understanding, and order in a complex and changing world (Frank, 2010). In qualitative research, narrative analysis encompasses various practices and strategies (Riessman, 2008). The core aim of narrative analysis is to capture the contextual, diverse, and nuanced nature of human experiences rather than to generalise (Blix et al., 2013). Stories shape how people perceive themselves, their relationships with others, and their capacity to act, reflecting the constituents of human reality (Frank, 2012).

The analysis model implemented in this study was inspired by Riessman’s (2008) narrative thematic analysis. Thematic narrative analysis aims to uncover the deeper meanings and messages embedded in narratives, enabling the identification of recurring themes across the dataset (Riessman, 2008). Thematic narrative analysis provides an in-depth exploration of helpers' self-perceptions and interpretations of reality.

The analysis, conducted by the first author and in collaboration with the third author, involved several phases. Firstly, possible narrative themes related to providing helpful support were identified by reviewing each interview individually, which involved reading transcripts, listening to audio recordings, and taking notes. Each narrative was then analysed individually, starting with thematic narrative analysis to determine its unique content (Riessman, 2008). A table was used to organise and code data relevant to the research question in each interview, identifying sequences that contained descriptions of helpful support. Next, the similarities and differences between the interviews were studied, and four themes were generated across the interviews. Based on this analytic work, a typology of thematic stories was created. Selected excerpts were woven into these descriptive narrative typologies, illustrating the different themes.

Four narrative themes emerged from the analysis: (1) the accessible helper, (2) the empathetic, acknowledging helper, (3) the caring, present helper, (4) the self-caring helper.

### Reflexivity and researcher positionality

The research team brought varied, practice‑based perspectives that likely shaped what we noticed and how we interpreted the data. The third author participated in the focus‑group interviews and led one of the three interviewing teams; the first author led the analysis and reviewed transcripts and audio recordings. The second and third authors reviewed analytic outputs and provided comments and analytical input, including independent readings of selected transcripts and discussion of alternative interpretations.

The third author has extensive prior research and practical experience with bereavement and next‑of‑kin issues in the substance‑use field; this experience supported sensitivity to grief‑related themes but may also have inclined our reading in particular directions. The other authors do not have prior research experience with bereaved populations but have long professional experience as helpers in Norwegian health and social services; their familiarity with local service structures and collegial practices contributed contextual insight.

We took pragmatic steps to increase transparency and to address possible interpretive tendencies and taken‑for‑granted assumptions: team discussions were used to compare readings, and brief analytic notes were kept. These steps were limited in scope and are reported to increase transparency about possible influences of researcher backgrounds on interpretation. We also note that the Norwegian service context, where statutory responsibilities for bereavement support and expectations of collegial practice are often taken for granted, may have shaped our interpretations and limited transferability to other settings.

### Ethical considerations and methodological assessments

Approval has been obtained from the Norwegian Agency for Shared Services in Education and Research (reference number 525501), which is the entity in Norway responsible for assessing whether research meets ethical and privacy requirements. Additionally, the project has been approved by the Western Norway University of Applied Sciences. Participants in focus group interviews were informed that their participation was voluntary and that they could withdraw up to one month after the data was collected, but not after the analysis had begun. No individual participants can be identified in the presentation of the findings. All project data were securely stored on the Western Norway University of Applied Sciences’ research server (#FOU-000124). Only essential data were included in the study.

All participants received written information about the study's purpose, methods, and procedures, which was reiterated verbally at the start of the interview. They were informed that data would be published anonymously, signed a consent form, and were assured of anonymity, confidentiality, and the right to withdraw at any time. Ethical considerations were carefully assessed and organised by the leadership of the main project. Given that the study addresses sensitive and emotional themes, it was crucial that the researchers involved had substantial experience working with vulnerable topics in social and health services.

Focus group studies can uncover collective opinions and provide insights into the context in which these opinions are formed (Halvorsen, 2008). This method acknowledges that experiences and perceptions often evolve through social interactions (Halvorsen, 2008). The primary objective of focus group interviews is to create a research environment with minimal researcher influence (Malterud, 2012). A common critique of this method is the challenge of maintaining balanced group discussions and avoiding overly similar perspectives among participants (Malterud, 2012). As with all focus group studies, the group format may have constrained participants’ willingness to share critical reflections on organisational shortcomings or sensitive work-related challenges. This potential limitation was taken into account during analysis, and we included quotes that represented both consensus and more nuanced or hesitant expressions. Both the interviews and subsequent analysis inevitably carry some degree of subjectivity, as the author's background influences the interpretation of the data.

## Findings

This study examined professional helpers' narratives about providing support to individuals bereaved by drug-related deaths. These narratives are shared from the perspective of the helpers. Four distinct narratives have been identified. Each narrative has been given a name, and while the narratives are interconnected, they have been separated analytically. Each highlights a specific theme. The excerpts from the interviews have been edited for clarity, and pseudonyms have been assigned to the narrators. The reflections associated with the quotes illustrate the author’s interpretation and understanding of the narratives (cf. Frank, 2010).

### Narratives of the accessible helper

The narratives of helpers providing support often start by emphasising the importance of being accessible and quick to establish contact with the bereaved individuals. For this reason, the theme has been titled: Narratives of the Accessible Helper. Stig observed the importance of low-threshold services for bereaved individuals:

They need to be easily accessible, and it should be a low threshold for contact, because many people need help right away, and maybe don’t an hour later. It has to be readily available. (Stig)

Stig noted that the key to helpful bereavement care lies in providing low-threshold services with easily accessible support. He stressed the importance of being available to bereaved individuals when they are ready, without delay. Stig’s narrative highlights the helper's responsibility to seize the moment when bereaved individuals reach out, providing support immediately upon establishing contact.

Miriam observed that accessibility depends on the bereaved individual's knowledge of available services. She also identified additional barriers:

But people die from drug-related causes that aren’t overdoses—sometimes their body just gives out because they’ve taken too much. And I think there's a lot of shame tied to that. I mean, when you look on the municipality’s website, who are you supposed to call? (Miriam)

Her account points to two related barriers. Stigma and shame can deter help‑seeking, while unclear service information can prevent those who do seek help from finding the right entry point. These mechanisms may operate independently or interact—stigma can reduce information‑seeking, and poor communication can compound the problem for those who try to reach out.

Helpers reported a positive shift in the accessibility of services, transitioning from the previous decision-based services to today's low-threshold approach. This change has made it easier for bereaved individuals to access support services. Many helpers express satisfaction with this, as it also made it easier to reconnect with the same helper, as noted by Maria:

Here, you can just walk through the door. You don't need a referral. [...] And it's up to the bereaved to define their own needs. There’s no one sitting there deciding whether your problem is big enough to receive help. It's actually up to them to say what they need when they come in, and we start from there. [...] When the case is closed, they have my phone number. (Maria)

Maria's statement portrays an open-door approach to municipal support services for bereaved individuals, without the need for a referral. No judgement is made about whether there is a legitimate need, whether it is the first or a subsequent visit. Stories from the helpers interviewed underline that useful help always starts with accessibility. No matter how good the help is, it does not matter if the person in need cannot find the help.

### Narratives of the empathetic, acknowledging helper

This theme involves narratives emphasising the importance of encountering an empathetic, acknowledging helper who recognises and validates bereaved people’s experiences. Being emotionally moved as helpers seems important both for acknowledging the deceased person's value as a human being and for giving the grieving recognition as persons in need of care. In several accounts, helpers described being emotionally moved by their interactions with bereaved individuals. Peder talked about his emotional experience during these encounters, as illustrated in the following excerpt:

I think that [...] when they present their stories and everything they’ve been through, it’s difficult, it really moves you. Sometimes, you just want to do something to help—like take them home with you or something, you know? Because there's a lot of crying, but also a lot of laughter, of course. It's touching, and it’s painful. (Peder)

Peder expressed a wide range of emotions, describing both joy and sorrow, inadequacy and meaningfulness, in his interactions with bereaved individuals. His account demonstrates the importance of managing not only the emotions of the bereaved but also the helper's own feelings. Despite the conflicting emotions, Peder described the encounters as deeply meaningful.

Several narratives highlighted the positive emotions that emerge in the interactions between helpers and bereaved individuals. Iselin, for example, spoke about the significance of familiarity in these relationships:

[...] but also with certain phone calls, when they say, ‘Wow, you still remember me?’ That feels so good. (Iselin)

Iselin expressed how rewarding it feels to hear such words from bereaved individuals, who are surprised that she still remembers them. Being remembered in the context of a helper-bereaved relationship can provide a sense of recognition and significance— for both the bereaved and the helper. Her account also suggests that the relationship between helpers and bereaved individuals extends beyond physical encounters.

Narratives of helpers who provide helpful support to bereaved individuals often reflect the helper’s role in these interactions. Harald’s narrative illustrates how an empathetic, acknowledging helper can seamlessly transition between being a professional and a fellow human being when supporting bereaved individuals.

[...] but we can't work with people and be professional all the time, because we bring our whole selves, and all our life experiences, into what we do. Sometimes they don't need a professional—they just need a fellow human being to be there with them. [...] These are bereaved people who’ve been under stress and grief for many years—they’re exhausted. (Harald)

Harald described the helper's ability to switch between being a professional and a human being, as well as the personal impact of these encounters.

### Narratives of the caring, present helper

This theme highlights the importance of helpers’ outward presence and public acts of care—attending funerals, representing institutions, and offering visible support that honours the deceased and supports the bereaved. Interviewees describe how such presence both signals respect and provides a starting point for ongoing contact. The first account comes from Maria, who spoke clearly about attending funerals:

As far as possible, someone from our team should attend the funerals of those we’ve worked with [...] It’s a matter of respect, and it's important to set aside the time, for so many reasons. It’s about representing the municipality, showing up. We know it means a great deal to many people, to have someone right there until the very end. (Maria)

Maria expanded on the significance of attending funerals, respectfully describing how, in addition to representing herself, the helper also represents society, which owes it to the deceased and the bereaved to attend the funeral.

For many helpers, the first meeting with bereaved relatives often takes place at the funeral. Attending the funeral and sharing the ritual allows helpers to learn about the deceased and the bereaved, providing a natural opening for later conversations. Harald described this process:

What I’ve noticed at funerals is that it feels good to be there, but more importantly, it’s helpful for us to be able to share the experience with the bereaved later. For example, you can say something like, ‘That was a beautiful story he told about your son.’ It’s a very good starting point and also resonates with us personally—it leaves an impact. (Harald)

Harald’s account illustrates how stories about the deceased can create a meaningful basis for subsequent dialogue that benefits both parties.

Narratives in this theme also address how a visible, caring presence can challenge stigma associated with certain causes of death. Iselin explained why attending funerals matters in this regard:

We ask to attend funerals because I think it's also a way for us to process everything we’re dealing with. Being there with them, hearing the kind words, and learning more about the boy or girl who passed—it brings out the person, hearing all that, seeing all the flowers, it’s part of the ritual, and it helps us handle the emotional weight. Just think about how a child is treated at school when their mother dies of an overdose compared to when she dies of cancer. Is this something we discuss openly? It’s a difficult challenge, no doubt. (Iselin)

Iselin points to the invisible label that can separate those bereaved by drug‑related deaths from other bereaved individuals. Moreover, she highlights how the helper’s public presence can help counteract that stigma. A caring, present helper thus affirms the value of both the deceased and their surviving relatives, signalling that they are seen and respected.

### Narratives of the self-caring helper

The narratives of helpers who provide helpful support underscore the importance of prioritising their own emotional well-being. By recognising that this work is both emotionally demanding and rewarding, helpers are better able to remain present for the bereaved and to sustain their role over time. Crucially, helpers emphasise that self‑care is not a task they can carry out alone: collegial support and organisational systems are necessary enablers of effective self‑care in practice.

Knut described routine practices for processing difficult encounters and the importance of having colleagues who will listen:

I think, at least for us, we’re pretty good at venting—sometimes even debriefing ourselves—after experiencing something that really affects us. I'd say we manage it well, and there's always space to sit down, talk, and get things off our chest. If it's me, there are people who will listen, and if it's someone else, we’re there for them. So I feel there’s plenty of opportunity to process these powerful experiences. (Knut)

Knut’s account illustrates individual self‑care practices (debriefing/venting) that occur within a supportive team context; everyday peer conversations create space for helpers to process strong feelings and thereby maintain their capacity to provide care.

Narratives of helpers needing to share their feelings and impressions highlight their role as recipients of stories shared by the bereaved. Fia emphasised that helpers “carry” others’ stories over time and that formal systems are needed to lighten this load:

Another important aspect, I think, is that we carry with us other people's stories. People share their lives with us, and being the one who listens and takes it all in can be challenging over time. That’s why it’s so important to have systems for guidance and support from colleagues, especially in a setting where you’re listening to people’s stories and carrying them with you. (Fia)

Fia’s description conjures the image of a helper carrying a backpack full of stories. Her account illustrates how individual self‑care relies on organisational frameworks: supervision, guidance, and structured debriefing opportunities help prevent emotional overload. As Fia noted, sharing these stories with colleagues makes it easier to carry them forward and to make room for new ones; sharing, therefore, appears to reduce the emotional burden.

Sharing these experiences and their impact can serve as a preventive measure, making it possible for helpers to continue their work, as Asbjørn explained:

We need to focus on prevention so we can stay strong for as long as possible. [...] I have complete trust in the people I work with because we work so closely together. That makes it possible to say and express whatever we need to, really. That's perhaps the most important thing, those everyday conversations. Especially right after it happens, you know? We’re there immediately after someone has passed. [...] Guidance is important, but support from your colleagues is everything. We don't talk so much about the case, but more about what it does to us. (Asbjørn)

Asbjørn links individual resilience to everyday collegial support and organisational practices that normalise emotional processing. Together, these narratives show that self‑care is not a private obligation but a practice embedded in team routines and workplace systems that enable helpers to remain effective and to sustain their roles.

## Discussion

The four key findings from the study are: (1) the accessible helper, (2) the empathetic, acknowledging helper, (3) the caring, present helper, and (4) the self-caring helper. Despite being presented as distinct themes, they are interconnected but analytically separate. Based on the research question, “How do professional helpers describe the key factors in providing helpful support to bereaved individuals?” the results will be discussed under two headings (that capture several elements across the identified themes): The Helpful and Helpless Helper and Helpful Self-Care, in order to highlight their interdependence and support a more comprehensive understanding of the dynamics involved.

## The helpful and helpless helper

Through their stories, helpers described the low-threshold services they provide, demonstrating a clear understanding of the importance of being accessible and responsive to individuals bereaved by drug-related death. However, despite good intentions and an “open-door” approach, there are still significant barriers to accessing support, owing to a lack of recognition and exacerbated by grief and stigma (Templeton and Yarwood, 2018; Dyregrov & Selseng, 2022). These barriers appear both before and after the death occurs (Reime et al., 2022). As a result, the support system continues to struggle with implementing services for bereaved individuals (Løseth et al., 2023). International research reports similar patterns—stigma, disenfranchisement, and unmet support needs suggesting that the challenges identified in Norway reflect cross‑national trends rather than being uniquely local (e.g., Lambert et al., 2022; O'Callaghan, 2024; Stout & Fleury-Steiner, 2023; Titlestad et al., 2021). Helpers in this study noted that individuals bereaved by drug-related death may hesitate to seek help if their needs are not perceived as legitimate. However, in this study, helpers placed less emphasis on the need for recognition as a distinct social group, focusing instead on providing easily accessible, low-threshold services for those bereaved by drug-related death. This aligns with research showing that individuals bereaved by drug-related death are not sufficiently recognised as service users by the support system. The Norwegian Institute of Public Health (2022) reports a significant increase in the use of healthcare services since 1998, alongside rising expectations and limited capacity. Taken together with international findings, this underscores that bereaved people should be explicitly recognised as service users and that both local, pragmatic responses (low‑threshold access) and broader policy measures (recognition, resources, and training) are needed to close systemic gaps. Recent studies show that this group is not sufficiently recognised within the support system (Kalsås et al., 2022; Templeton and Yarwood, 2018). Indeed, the opposite of recognition is marginalisation (Honneth, 2008). According to Honneth, (2008), recognition is a fundamental psychological need and a prerequisite for personal development and the ability to utilise one's resources in a positive way. Similarly, Maslow, (1943) describes this as the experience of being valued, respected, and recognised by others, both individually and as part of a group.

At the individual level, the helpers told a different story. They described providing helpful support by recognising bereaved individuals through conversation, focusing on compassion and allowing themselves to be touched by their stories. A respectful, safe, and compassionate approach is necessary for bereaved individuals to accept support (Dyregrov & Selseng, 2022). Being recognised by a helper can reduce feelings of marginalisation caused by social stigma, fostering a greater sense of freedom for the bereaved (Dyregrov & Selseng, 2022). Moreover, helpers emphasised being mindful of their role, balancing professionalism with compassion. These findings make it clear that helpers need to understand bereaved individuals both as a collective group and on a personal level. Helpers must be conscious of how they engage with bereaved individuals, including appropriate language, kindness, respect, and knowledge regarding traumatic grief (Løseth et al., 2023). This aligns with Honneth (2008) view that people have a fundamental need for recognition through kindness, which is often expressed in caregiving relationships. A helper's kindness and compassion can also be seen as an altruistic act (Trivers, 1971), as they connect with the experiences and emotions of the bereaved. A deeply empathetic helper maintains a strong connection to their own emotions and bodily experiences (Amlund, 2008). Through empathy, the helper gains direct insight into the emotional state of the bereaved, allowing them to feel seen and recognised in their situation (Amlund, 2008).

## Helpful self-care

Understanding the helper's needs within their role is essential. A key finding from the study is the helpers' accounts of attending funerals, which shed light on several important aspects of support: (a) respect for the deceased, (b) respect for and connection with the bereaved, (c) participation as a response to social neglect, and (d) the helper’s own need to grieve, as they may also experience loss. Across all six focus group interviews, the helpers emphasised the important role of peer support among colleagues, describing it as indispensable. A helper's ability to balance their personal and professional roles relies on self-awareness of their needs and limitations (Germer & Neff, 2013). Seeking support from colleagues is an expression of self-compassion (Neff & Dahm, 2015). Being mindful of one's emotional state can help prevent caregiver burnout (Isdal, 2017). Peer conversations often serve as a form of supportive environment for helpers, where they can speak freely and without interruption (Goffman, 1971). In addition to providing emotional support, these conversations contribute to professional insight and problem-solving (Jørgensen, 2016). In this respect, peer support not only plays a preventive role but can also have an impact on measures (Jørgensen, 2016). The success of peer support and prevention efforts depends on workplace structures. It requires organisational systems and culture that recognise the value and need for peer support and debriefing. It can be argued that most individuals working in support services possess altruistic instincts, which can be described as an innate inclination towards sympathy for others (Trivers, 1971). Experiencing meaning is essential for developing a sense of coherence—that life is emotionally comprehensible and that things or people are worth the effort and commitment (Antonovsky, 2012).

## Strengths and limitations of the study

This study, involving 29 participants, benefits from extensive and detailed interviews, providing a rich source of material. Narrative analysis was considered appropriate for exploring helpers' perspectives on helpful support, offering valuable insight into their experiences in the role. The inclusion of several quotes ensures transparency and validity, allowing readers to evaluate the themes more effectively. The stigmatised nature of drug‑related death is an important contextual limitation: public and professional stigma may shape which cases helpers recall, how they describe them, and which practices they consider acceptable to report. Given the strong anchoring of bereavement support within the Norwegian municipal welfare model, the transferability of the findings to other national contexts may be limited. However, the challenges described, such as stigma, fragmented services, and uncertainty around responsibility, are also widely reported in international research, suggesting that several of the mechanisms identified may be relevant beyond Norway. The limitations of the study also reflect the common challenges associated with focus groups. Focus‑group dynamics may also have increased socially desirable responding and limited participants’ willingness to discuss organisational shortcomings or bad practice. There was no guarantee that these participants were the most experienced professionals within their respective municipalities. All data is taken from the participants' experiences, which may reflect variable levels of direct experience with drug‑related bereavement, assumptions, and interpretations. Among the authors, only the third author has direct experience working with individuals bereaved by drug-related deaths. However, all the authors have backgrounds in health and social services. This can be considered both a strength and a limitation. We recorded reflections throughout the research process and used team discussions to surface and challenge preconceptions (cf. Gilje & Grimen, 2007).

## Concluding remarks and implications for praxis

The results of the study indicate that helpers have a solid understanding of the needs of bereaved individuals. Their insights provide important contributions to the knowledge base regarding drug-related bereavement and the support needs of those affected, as well as guidance on providing helpful assistance. Helpers consider accessibility a key factor in delivering low-threshold services. Moreover, they place a strong emphasis on personal qualities such as recognition and compassion in their roles while also highlighting the importance of self-care and peer support among colleagues to facilitate compassionate help. However, the Norwegian Directorate of Health's (2017) guidelines are neither widely known nor well integrated into municipal services. Consequently, they provide limited direction for support services and offer little help to those bereaved by drug-related death. The discrepancy between the perspectives of helpers and the needs of the bereaved highlights several areas that require attention. Further research is needed to examine how health and welfare services perceive the accessibility and content of services for bereaved individuals. There is a need for greater insight into the resources available to professional helpers, the barriers and opportunities they face, and their capacity to meet the needs of the bereaved. It is also important to develop a better understanding of stigma and attitudes, both within society and at an individual level. This knowledge is necessary to improve the accessibility and quality of support. Strengthening education and the quality of health and social services is crucial, as is recognising those bereaved by drug-related death as a distinct group. Although this study was conducted in Norway, a small social-democratic welfare state, the study addresses an international phenomenon in which the group’s challenges extend beyond national contexts.
